# Novel APD Array Configurations for Improved Detection Area and Frequency Response

**DOI:** 10.3390/s25061671

**Published:** 2025-03-08

**Authors:** Xuan Zeng, Xuzhen Yu, Hewei Zhang, Yi Lu, Yanli Zhao

**Affiliations:** Wuhan National Laboratory for Optoelectronics, Huazhong University of Science and Technology, Wuhan 430074, China; m202273384@hust.edu.cn (X.Z.); m202373363@hust.edu.cn (Y.L.)

**Keywords:** avalanche photodiode, large detection area, gain peaking, signal-to-noise ratio

## Abstract

This paper presents two novel avalanche photodiode (APD) array structures designed to significantly enhance both detection area and bandwidth, overcoming the common trade-off between these parameters in conventional photodetectors. The impact of various parameters on the bandwidths of the two distinct array structures was theoretically simulated. Experimental validation using the self-fabricated 2 × 2 array on PCB board confirmed the bandwidth enhancement realized through inductor integration, with one APD array demonstrating an increase to 780 MHz (1.41 times greater) and another showing an increase to 1.21 GHz (1.35 times greater). Unlike prior works where array bandwidth is often lower than single detectors, our structures maintain high bandwidth while expanding the detection area. Structure 2 is particularly recommended over Structure 1 because of its lower noise, better signal-to-noise ratio (SNR), and reduced power consumption.

## 1. Introduction

Developing high-performance photodetectors with both large detection areas and high-speed responses remains a critical challenge in contemporary optoelectronics. With the growing demand of higher data rates and larger detection areas in applications such as free space optical communication [1,2,3,4,5,6], lidar [7,8,9], and medical imaging [10,11], the performance requirements for photodetectors have become increasingly stringent. Recent advancements in material systems and device architectures have opened new avenues for optimizing photodetector performance [6,12]. Unfortunately, traditional photodetectors tend to face a trade-off between the expanded detection areas and bandwidth. To overcome this limitation, high-speed photodetector arrays are strongly desired instead of single photodetectors with large detection areas [13,14,15].

To date, research on single-output photodetector arrays with large detection areas has been scarce. One study introduced a Si/Ge avalanche photodiode (APD) array that utilized pixel interconnects, achieving a bandwidth of 142 MHz for each pixel, which was reduced to 52 MHz post-cascading [16,17]. In another instance, a Si/Ge APD array with a pixel diameter of 33 μm provides a 9 GHz bandwidth for a single APD, with a gradual decrease to 6.7 GHz, 1.2 GHz, 0.7 GHz for 2 × 2, 5 × 5, 10 × 10 arrays, respectively [18]. A 4 × 4 PIN PD array designed for optical wireless receivers has been reported to have a bandwidth of 1 GHz per PD, which is reduced to 610 MHz after packaging [19]. Furthermore, an InAlAs/InGaAs APD array utilized as a position-sensitive detector in free-space optical communication maintains a bandwidth of 1.8 GHz for a single APD, which diminishes to 1 GHz when configured as a 3 × 3 array [5]. A high-speed 8 × 8 InGaAs/InP PIN array for free-space optical systems, with a pixel diameter of 40 μm and the detection area of 0.4 mm × 0.4 mm, achieves a uniform bandwidth of 8.5 GHz across the 8 × 8 array, matching the bandwidth of the individual pixels [14]. For the array discussed, as array size increases, the bandwidth tends to decrease or remain constant without additional measures to improve it.

While inductive peaking has been extensively studied for single photodetectors [20,21,22,23], its application to photodetector arrays remains unexplored. Traditional photodetector arrays often suffer from bandwidth degradation as the detection area increases, limiting their performance in high-speed application. This paper addresses this challenge by proposing two novel APD array structures that not only maintain a large detection area but also achieve significant bandwidth enhancement through strategic inductor integration. In the simulation section, two diverse array structures are proposed and the influence of array size, parallel resistors, and inductors on the array bandwidth of both array types is investigated. In the experimental section, exploratory 2 × 2 arrays for two distinct structures are fabricated using two APDs with different absorption layer thicknesses. The arrays were tested for bandwidth, power consumption and signal-to-noise ratio to provide a comprehensive evaluation of their performance. This work may provide new perspectives for achieving higher bandwidths in large-scale arrays.

## 2. Structures of Two-Dimensional Photodiode Arrays

The fundamental aim of a two-dimensional photodiode array is to enlarge the detection area without degrading the bandwidth, which is a critical requirement for high-speed optoelectronic applications. As the detection area of an individual photodetector expands, the junction capacitance $C_{j}$ increases, leading to an extended RC time constant and resulting reduction in the bandwidth. To address the trade-off between bandwidth and detection area, a single large-area photodetector was transformed into an array composed of multiple smaller photodetectors. The key is to interconnect these small detectors such that the bandwidth of the array remains unaffected by the enlargement of the detection area.

**Figure 1:**
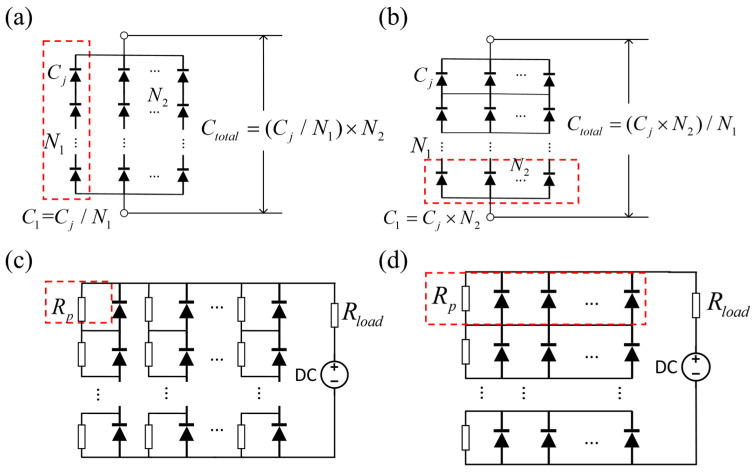
Two different array structures: (**a**) Structure 1; (**b**) Structure 2. The red dashed boxes in (**a,b**) highlight the connection sequence of detectors. To prevent the obstruction of the electrical current path, (**c**,**d**) picture the advancement implemented in Structure 1 and Structure 2 through the inclusion of parallel resistors. The red square highlights how parallel resistors are added differently in each structure: in Structure 1, a parallel resistor is added to each detector, while in Structure 2, a parallel resistor is added to each row of detectors.

Figure 1 illustrates two distinct array structures: one where pixels are initially connected in series and then in parallel (Structure 1, see Figure 1a), and another where the order is reversed, connecting in parallel before series (Structure 2, see Figure 1b). When the array has an equal number of rows and columns (i.e., $N_{1}=N_{2}$), the total capacitance of both structures is equivalent to that of a single photodetector. Consequently, both array designs have the potential to expand the detectable area, while maintaining a high-frequency response.

In practical applications, there is a possibility that the incident light spot may not fully cover the entire photodetector array. This can lead to photodetectors in the non-illuminated regions remaining in a high-resistance state, effectively interrupting the photocurrent flow. To avoid this scenario, parallel resistors ($R_{p}$) were introduced into both array structures to establish a path for the photocurrent (see Figure 1c,d).

## 3. Theoretical Simulation and Analysis of Photodetector Arrays

### 3.1. Array’s Bandwidth

**Figure 2:**
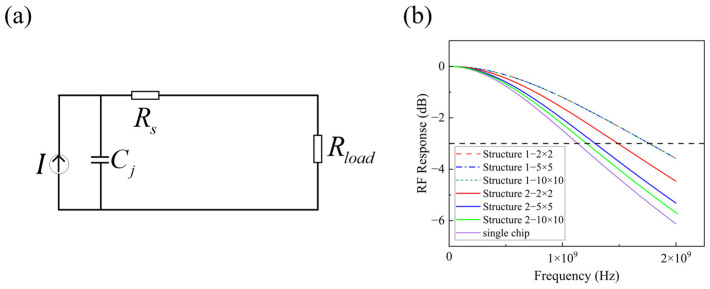
The circuit diagram for (**a**) single chip and (**b**) the variation in bandwidth with the size of the array for two structures. The horizontal dashed line indicates the frequency at which the RF response drops by 3 dB, defining the system’s bandwidth.

In standard photodetector applications, a fixed load resistor of 50 Ω is added (referred to as a single chip, see Figure 2a). For both array structures, the effect of the array size (N × N) on the bandwidth was initially analyzed. Assuming a junction capacitance of 2 pF, the series resistor $R_{s}$ for a single photodetector was set to 20 Ω, with the array’s parallel resistor $R_{load}$ at 50 Ω in the simulations. As shown in Figure 2b, for Structure 1, the array bandwidth is independent of the array size, consistently exceeding the bandwidth of a single photodetector. In the case of Structure 2, the bandwidth shows a gradual decline with an increase in the array size, but it still remains higher than the bandwidth of a single chip. Under the condition of an equal array size, Structure 1 has a higher bandwidth than Structure 2. This is primarily because Structure 1 introduces a smaller additional resistance compared to Structure 2.

**Figure 3:**
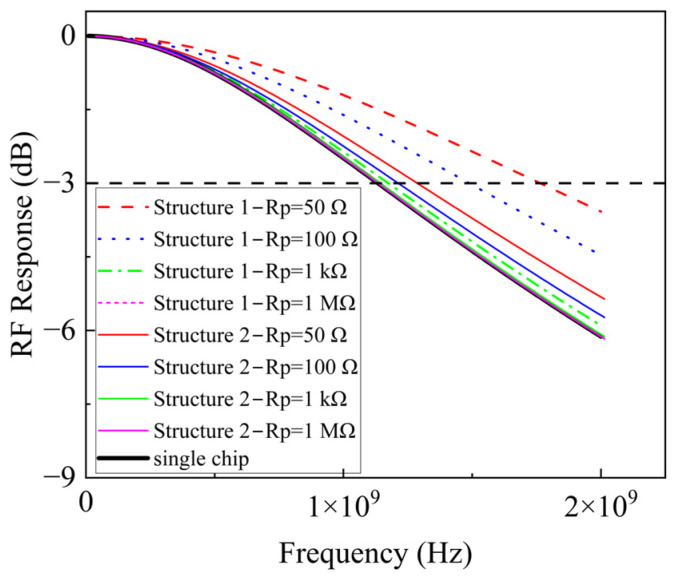
The array bandwidth varies with $R_{p}$.

In addition to the array size, it is important to consider the influence of the $R_{p}$ values on the array bandwidth. A 5 × 5 array served as an example in the simulation that investigated the bandwidth variation with different $R_{p}$ values across both array structures. As shown in Figure 3, the bandwidths for both structures decreased as the $R_{p}$ increased. When the $R_{p}$ was much higher than the load resistor (50 Ω), the bandwidth for both structures was the same as that of a single chip.

### 3.2. Optimization of the Array Structure

**Figure 4:**
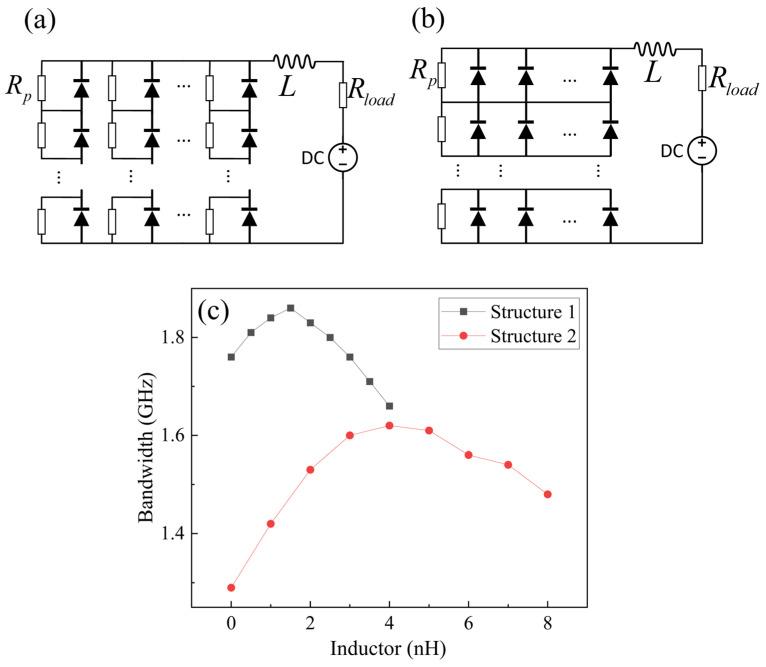
Schematic diagram of the circuit with an inductor added at the end of the array. (**a**) Structure 1, (**b**) Structure 2, and (**c**) the graph of the array bandwidth changing with the inductor’s value.

The bandwidth of a single photodetector can be enhanced by integrating it with an inductor, transforming its equivalent circuit from a simple RC circuit to an RLC circuit [22,23,24]. To raise the array bandwidth, the introduction of inductors into the array structure is considered. First, an inductor was added at the end of the array to control the bandwidth, as shown in Figure 4a,b. The bandwidths for both array structures were simulated with $C_{j}$ = 2 pF, $R_{s}$ = 20 Ω, $R_{p}$= $R_{load}$ = 50 Ω, and the resulting graphs showing how the bandwidth varies with inductance are presented in Figure 4c.

By employing this approach, the bandwidth of Structure 1 can be enhanced from 1.76 GHz to 1.86 GHz, an increase of 1.06 times. Similarly, for Structure 2, the bandwidth can be improved from 1.29 GHz to 1.62 GHz, which is a 1.26-fold increase. Thus, the influence of the inductor on bandwidth optimization is more effective in Structure 2 than Structure 1. However, it does not surpass the optimal bandwidth enhancement achieved by a single detector integrated with an inductor, which is 1.44 times greater [20].

**Figure 5:**
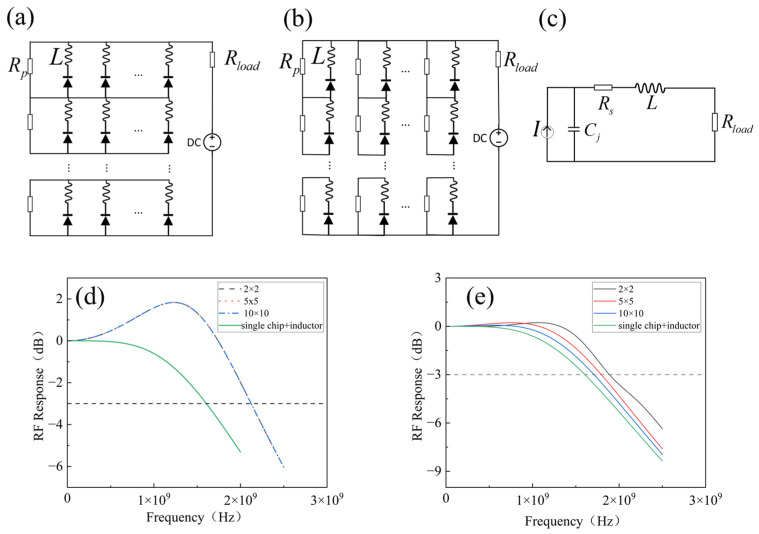
Schematic diagram after introducing an inductor into each cell of the array: (**a**) Structure 1 and (**b**) Structure 2. The circuit diagram for (**c**) single chip + inductor. Graph of the array bandwidth variation with array size after the introduction of inductors: (**d**) Structure 1 and (**e**) Structure 2.

Another approach for placing inductors within the array is to add an inductor to each cell within the array to control its bandwidth. The structures of both arrays are illustrated in Figure 5a,b. The circuit configuration for tuning the bandwidth of a single photodetector with an inductor (single chip + inductor) is shown in Figure 5c. When changing the size of the array, the simulation settings remained the same, with $C_{j}$ = 2 pF, $R_{s}$ = 20 Ω, $R_{p}$ = $R_{load}$ = 50 Ω. It can be observed that Structure 1 maintains a constant bandwidth (See Figure 5d). For Structure 2, the bandwidth decreased with the expansion of the array, gradually approaching the bandwidth of the single chip + inductor scenario (See Figure 5e).

**Figure 6:**
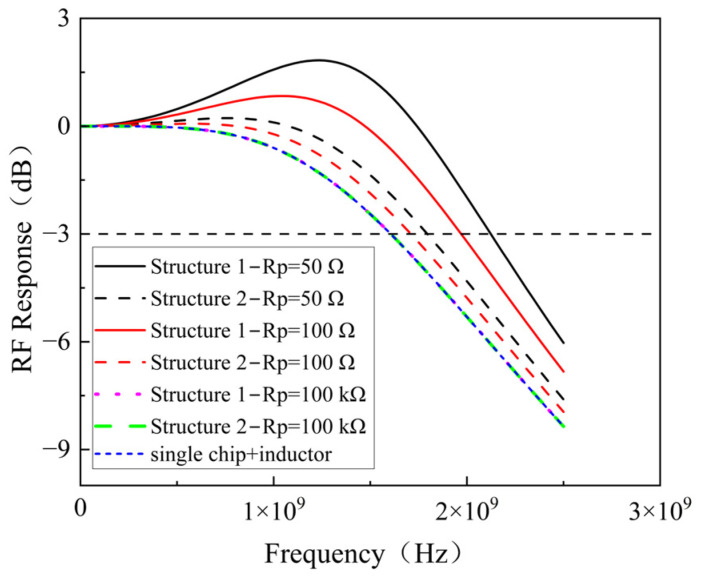
Bandwidth curves of the two arrays at different $R_{p}$.

Furthermore, following the introduction of inductors, the impact of varying the $R_{p}$ on the bandwidth of both array structures was studied. As depicted in Figure 6, for both structures, an increase in $R_{p}$ corresponds to a reduction in bandwidth. With a smaller value, Structure 1 has a greater bandwidth than Structure 2. However, when $R_{p}$ is much larger than the fixed 50 Ω load resistance, the bandwidth of both Structure 1 and Structure 2 match, becoming equivalent to the bandwidth of a single chip + inductor.

Although the array can achieve a higher bandwidth when $R_{p}$ is set to 50 Ω, in actual applications, power consumption issues must also be considered. Numerous parallel resistors of the array may result in considerable power loss. For an APD operating at a higher reverse bias voltage, it is advisable to use a resistor with a higher resistance value for $R_{p}$. This strategy enables the array to maintain a bandwidth exceeding that of a single chip while simultaneously maintaining a lower level of power consumption.

## 4. Experimental Results of Inductor-Integrated APD Arrays

### 4.1. APD Parameter Extraction

Optimizing the bandwidth of photodetectors equipped with inductors requires precise determination of the photodetector’s series resistance $R_{s}$. The ideal inductance can be calculated as follows [20]:

(1) $$L=\frac{C_{j}{\left(R_{s}+R_{load}\right)}^{2}}{2},$$

**Figure 7:**
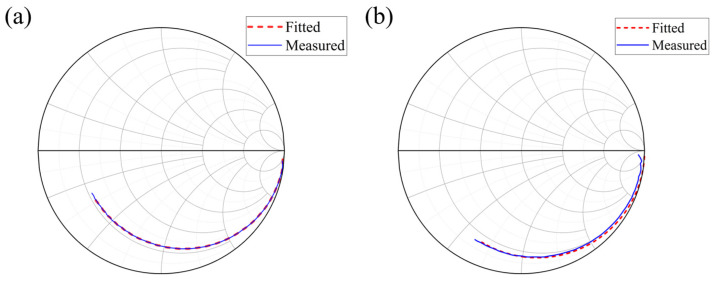
Measured (blue line) and fitted (red line) $S_{22}$ curves for (**a**) the thinner APD and (**b**) the thicker APD.

Typically, the values of $C_{j}$ and $R_{s}$ can be obtained by measuring the scattering parameters ($S_{22}$) of the photodetector and fitting them to its equivalent circuit model [25,26]. Two InGaAs/InAlAs APDs with a diameter of 200 μm and a uniform structure but varying absorption layer thicknesses developed in our laboratory are referred to as the thinner APD (1 μm) and the thicker APD (2 μm) [27]. Figure 7a,b shows the measured and fitted $S_{22}$ curves (from 20 MHz to 1 GHz), respectively. For the thinner APD, $C_{j}$ and $R_{s}$ are extracted to be 5.01 pF and 10.98 Ω; for the thicker APD, $C_{j}$ and $R_{s}$ are extracted to be 2.79 pF and 12.60 Ω.

With the fitted values of $C_{j}$ and $R_{s}$, the most effective inductance for the thinner APD and the thicker APD can be calculated to be 9.3 nH and 5.5 nH, respectively, as determined by Equation (1). Because of the absence of inductors with these exact values in our laboratory, an 8.5 nH inductor was used for the thinner APD, and a 7 nH inductor was utilized for the thicker APD in the follow-up experiments.

### 4.2. Array Fabrication

**Figure 8:**
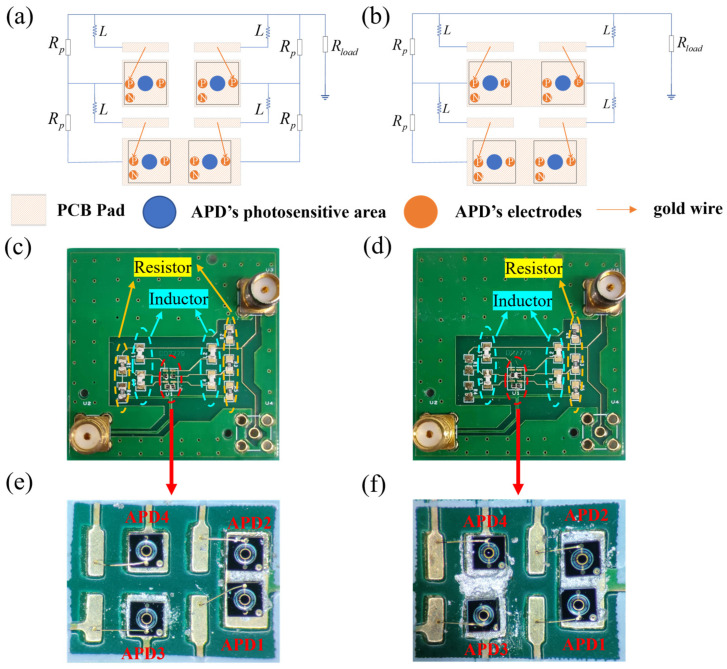
The schematic diagrams of the PCB boards for (**a**) Structure 1 and (**b**) Structure 2. The physical images of the PCB boards for (**c**) Structure 1 and (**d**) Structure 2. The actual gold wire bonding diagrams of APD arrays for (**e**) Structure 1 and (**f**) Structure 2.

To verify the feasibility of the two array structures, 2 × 2 arrays for both structures were fabricated by externally integrating resistors and inductors on a PCB board. Figure 8a,b is the schematic diagram of the PCB boards for Structure 1 and Structure 2, respectively.

The APD chips were equipped with a single N-electrode at the back and a pair of P-electrodes alongside another N-electrode at the front. Attachment of these chips to the PCB pads was achieved through the application of conductive silver adhesive. This adhesive not only ensured the stable positioning of the chips but also established an electrical link with the N-electrodes, ensuring proper functioning within the structure. Additionally, gold wire bonding was employed to link the P-electrodes to the pads. Figure 8c,d depicts the actual PCB boards corresponding to the two array structures, where Figure 8e,f shows the physical sections of the chips for both structures. It should be noted that despite the variations in thickness, the thinner and thicker APDs used in the experiment were visually identical.

### 4.3. Power Consumption and Frequency Response

Considering the high operating voltage of the APD, with the thinner APD operating at 28 V and the thicker APD at 45 V, to reduce the power consumption, an $R_{p}$ value of 500 kΩ was utilized in the experiments. During operation, the thicker 2 × 2 arrays consumed 18.5 mW for Structure 1 and 9.2 mW for Structure 2. On the other hand, the thinner 2 × 2 arrays used 6.3 mW for Structure 1 and 3.1 mW for Structure 2. The power consumption of these arrays was considerably lower than that array reported previously [14]. Compared with Structure 1, Structure 2 reduced the array’s power consumption owing to its fewer resistors.

**Figure 9:**
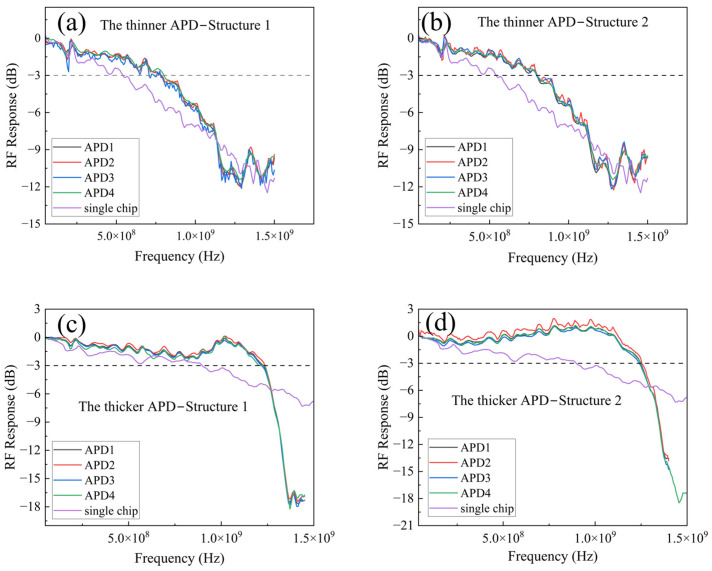
Bandwidth testing for the two types of APDs was performed in the 2 × 2 array structures of Structure 1 and Structure 2. The bandwidth of “APD1-4” were obtained from the four APDs within the array, and “single chip” refers to the bandwidth measured from an individual APD. For the thinner APD, the test result from (**a**) Structure 1 and (**b**) Structure 2; for the thicker APD, the test result from (**c**) Structure 1 and (**d**) Structure 2.

During the testing process, the incident light was focused onto a single APD within the array, and bandwidth measurements were performed on all the four APDs to verify the uniformity of the array. The bandwidth curves derived from the experiments are depicted in Figure 9, and the detailed data are listed in Table 1. The test results reveal that both the thinner and thicker APDs demonstrate satisfactory uniformity across the four APDs within the two array structures, and the bandwidths of Structure 1 and Structure 2 are consistent. It can be inferred that the results of the experiments correspond to those of the simulations.

Specifically, for the thinner APD, the single chip bandwidth is 556 MHz, while the bandwidth for the 2 × 2 arrays in both Structure 1 and Structure 2 ranges from 778 to 790 MHz, about a 1.41-fold increase over the single APD. The thicker APD’s individual bandwidth is 910 MHz, with array bandwidth ranging from 1.21 to 1.25 GHz, about a 1.35 times enhancement compared to the single APD.

**Figure 10:**
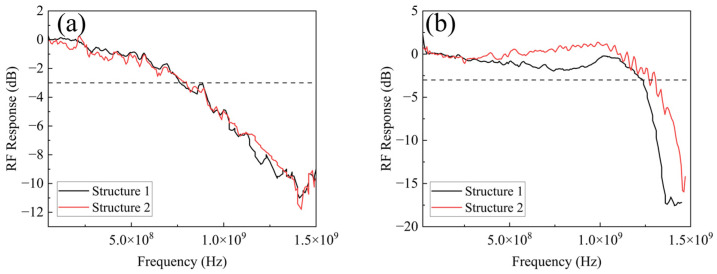
The array bandwidth result for the (**a**) the thinner APD and (**b**) the thicker APD of the 2 × 2 array when all APDs in the array are illuminated.

In practical applications, incident light may illuminate either a single pixel or multiple pixels within the array. To further strengthen the experimental validation, a new set of measurements was performed. A fiber collimator was used to transform the divergent spatial light from the fiber output into collimated light. The beam diameter was 4.0 mm, which was large enough to fully cover all four APDs in the 2 × 2 array. Based on this, the bandwidth of the array was tested. The test results are shown in Figure 10.

For the thinner APD, Structure 1 achieved a bandwidth of 775 MHz, while Structure 2 reached 790 MHz. For the thicker APD, the bandwidths of Structure 1 and Structure 2 were measured to be 1.23 GHz and 1.27 GHz, respectively. These values align closely with the bandwidths measured under single-APD illumination, conclusively demonstrating that the proposed array structures enhance the entire array’s bandwidth. This further validates the effectiveness of the design in maintaining high-speed performance while expanding the detection area.

### 4.4. Signal-to-Noise Ratio

The Signal-to-Noise Ratio (SNR) is a crucial parameter for receivers and was measured using similar methods previously reported in our laboratory [27]. The noise performance of the 2 × 2 APD arrays with varying absorption layer thicknesses, is presented in Table 2. Structure 2 consistently exhibits reduced noise levels compared to Structure 1 for both APD types. This reduction in noise is largely owing to the simplified design of Structure 2, which employs a smaller number of resistors, leading to a decrease in circuit noise.

**Figure 11:**
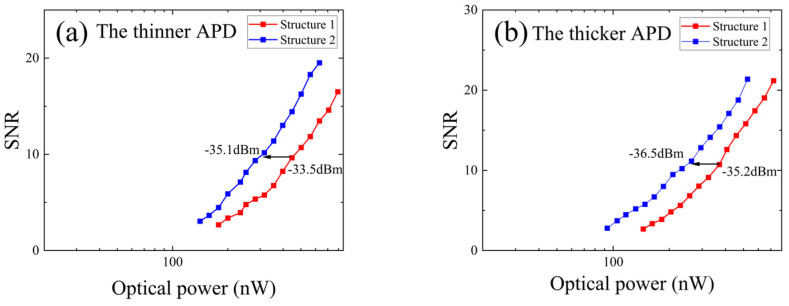
The relationship between the SNR and optical power for (**a**) the thinner APD and (**b**) the thicker APD.

The SNR was calculated by comparing the output signal pulse amplitude with the measured noise amplitude. These comparisons were made in response to a series of narrow signals from a signal generator at varying light power levels. The relationship between the SNR and input light power for the two types of APDs and their different arrays was assessed, and the results are presented in Figure 11. At an SNR of 10, the optical power required for Structure 1 is −33.5 dBm for the thinner APD, surpassing that of Structure 2 by 1.6 dB. Similarly, for the thicker APD, Structure 1 demands an optical power of −35.2 dBm, which is 1.3 dB more than Structure 2. Thus, for both types of APDs, Structure 2 exhibits a higher SNR than Structure 1 under equivalent incident light power, primarily because of the lower noise levels in Structure 2.

For the photodetector array, the bandwidth, power consumption, and signal-to-noise ratio performance all need to be considered in practical applications. While Structure 1 has higher bandwidth than Structure 2 when the parallel resistance $R_{p}$ is small, larger $R_{p}$ values are preferred in practice to reduce power consumption. Under this condition, the bandwidths of both structures become similar. Structure 2 is recommended due to its lower power consumption and better SNR performance, resulting from fewer resistors in the circuit.

**Figure 12:**
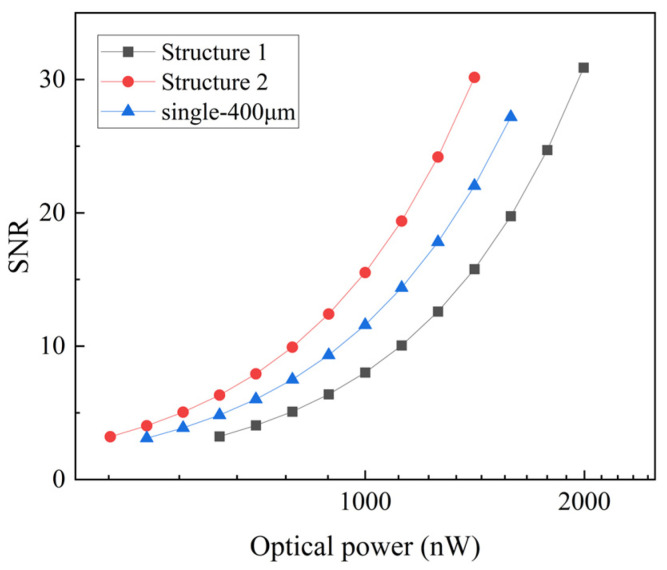
The relationship between the SNR and optical power for Structure 1, Structure 2, and single APD.

**Figure A1:**
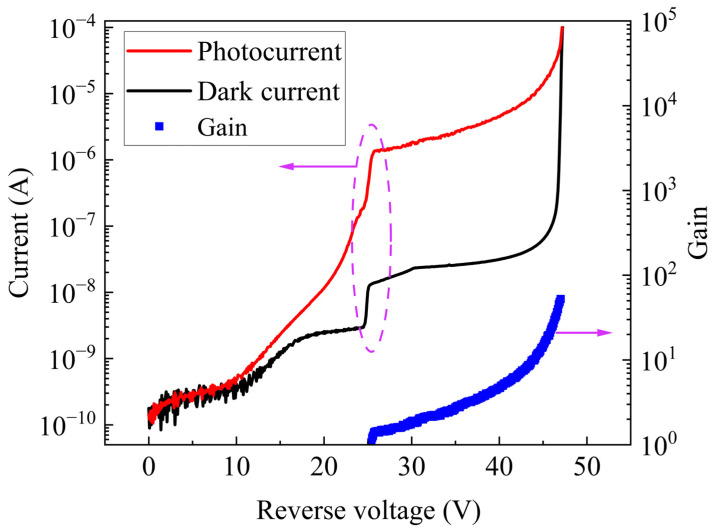
Photocurrent, dark current, and gain of the thicker APD.

The array device will ultimately be utilized as a standalone device. Consequently, it is necessary to compare the APD array with a single APD of identical detection area. Due to the absence of a single APD with a photosensitive diameter of 400 μm and similar structure, we consider simulating the SNR performance of a 2 × 2 array composed of four APDs, each with a photosensitive diameter of 200 um, and a single APD with a 400 um diameter. The simulation parameters are based on the performance characteristics of the thicker APD, as measured in experiments, such as a responsivity of 0.9 A/W, a gain of 10 (see Appendix A and Figure A1), and an avalanche coefficient of 0.27. As shown in Figure 12, the single APD exhibits inferior SNR performance compared to Structure 2. Considering that the detector can be modeled as an RC circuit, which attenuates signals, the transfer function is given by the following equation, where f represents the signal frequency and B denotes the detector bandwidth.

(2) $$\left|H(f)\right|=\frac{1}{\sqrt{1+{\left(f/B\right)}^{2}}}$$

Given that the 2 × 2 array has a broader bandwidth compared to the single APD, the signal attenuation by the single APD intensifies as the frequency of the transmitted signal rises. As a result, the SNR of the single APD declines more quickly than that of the detector array.

## 5. Conclusions

In this study, two novel photodetector array structures were designed, fabricated, and comprehensively characterized, and these structures successfully overcome the traditional trade-off between detection area and bandwidth. Through the integration of inductors, for the thinner and the thicker APD, the 2 × 2 arrays achieved a bandwidth increase of 1.41 times and 1.35 times, respectively, compared to their single APD. Especially, low power consumption was achieved for both array structures, with the power consumption for the two APDs in Structure 1 being 18.5 mW and 6.3 mW, and for Structure 2 being 9.2 mW and 3.1 mW, respectively. Moreover, the SNR performance of Structure 2 is superior to Structure 1 and that of a single APD with the same detection area. Based on these results, Structure 2 is particularly recommended because of its simplified circuitry, lower power consumption and superior SNR performance, making it a more advantageous structure for large-area photodetector arrays. These findings indicate the potential application value of these new detector arrays in high-speed free space optical communications.

Although the experimental section only verifies the performance of the 2 × 2 arrays, it is inferred from the simulation results that these structures are also applicable to larger and higher speed arrays. The array device in this paper is a combination of independent devices. Future research will investigate integrating spiral inductors on photodetector electrodes with photodetector arrays on a single chip to enhance the duty cycle and enable miniaturization of the array. Furthermore, the integration of lenses will be considered to enhance the responsivity of the array.

## Figures and Tables

**Table 1:** Detailed data for the bandwidth tests of the two APDs in both Structure 1 and Structure 2.

Chip Type	Single Chip	Array Structure	APD1	APD2	APD3	APD4
Thinner APD	556 MHz	Structure 1	782 MHz	785 MHz	783 MHz	778 MHz
		Structure 2	789 MHz	792 MHz	787 MHz	790 MHz
Thicker APD	910 MHz	Structure 1	1.23 GHz	1.24 GHz	1.21 GHz	1.21 GHz
		Structure 2	1.25 GHz	1.25 GHz	1.24 GHz	1.23 GHz

**Table 2:** Noise test detailed data for 2 × 2 arrays of two APD structures.

Chip Type	Structure 1	Structure 2
Thinner APD	7.11 mV	4.92 mV
Thicker APD	7.46 mV	5.38 mV

## Data Availability

All the data supporting this study are available. Additional data related to this paper are available from the corresponding authors upon request.

## Abbreviations

The following abbreviations are used in this manuscript:

APD	Avalanche photo diode
SNR	Signal-to-noise ratio

## Appendix A

The dark current and photocurrent of the thicker APD were measured, with the photocurrent measured at an incident optical power of 1 µW. The measured photocurrent and dark current data were processed to further determine the gain of the APD. As shown in the figure below, when the gain of the APD is equal to 1, the photocurrent is 0.9 µA, indicating that the responsivity of the APD is 0.9 A/W. Additionally, at an operating voltage of 45 V, the gain of the APD is 10.
